# Cellulose Nanopaper Cross-Linked Amino Graphene/Polyaniline Sensors to Detect CO_2_ Gas at Room Temperature

**DOI:** 10.3390/s19235215

**Published:** 2019-11-28

**Authors:** Hanan Abdali, Bentolhoda Heli, Abdellah Ajji

**Affiliations:** 1 NSERC-Industry Chair, CREPEC, Department of Chemical Engineering, Polytechnique Montréal, P.O. Box 6079, Station Centre-Ville, Montreal, QC H3C3A7, Canada; hanan.abdali@polymtl.ca (H.A.); bentolhoda.heli@polymlt.ca (B.H.); 2 Ministry of Education, P.O. Box 225085, Riyadh 11153, Saudi Arabia

**Keywords:** cellulose nanopaper, polyaniline, nanocomposites, functionalized graphene, CO_2_ sensing

## Abstract

A nanocomposite of cross-linked bacterial cellulose–amino graphene/polyaniline (CLBC-AmG/PANI) was synthesized by covalent interaction of amino-functionalized graphene (AmG) AmG and bacterial cellulose (BC) via one step esterification, and then the aniline monomer was grown on the surface of CLBC-AmG through in situ chemical polymerization. The morphological structure and properties of the samples were characterized by using scanning electron microscopy (SEM), and thermal gravimetric analyzer (TGA). The CLBC-AmG/PANI showed good electrical-resistance response toward carbon dioxide (CO_2_) at room temperature, compared to the BC/PANI nanopaper composites. The CLBC-AmG/PANI sensor possesses high sensitivity and fast response characteristics over CO_2_ concentrations ranging from 50 to 2000 ppm. This process presents an extremely suitable candidate for developing novel nanomaterials sensors owing to easy fabrication and efficient sensing performance.

## 1. Introduction

It is commonly regarded that high sensitivity, fast response and recovery times, as well as excellent selectivity and functionality at room temperature are important parameters for the evaluation of gas sensors [1,2]. Correspondingly, in the field of material sciences and chemical engineering, the quest to discover advanced materials with excellent performance is perpetual and immediate [3,4,5]. In the last few decades, research into functional materials with special nanoscale architecture has attracted great interest and has presented enhanced properties in numerous applications. These include energy storage application [6,7,8], catalysis application [9], medical applications [10], and gas sensing [3,11]. For gas sensing applications in particular, functionalized graphene-based, gas sensing materials have been prominent and, as a result, the subject of much research, because of its large surface area, unique mechanical, optical, thermal, magnetic, and electrochemical properties, and its variable conductivity, which makes it available for electron transport phenomena with very high electrical mobility, in the presence of oxidizing and reducing gases [5,11,12].

Polyaniline is commonly used in gas sensor materials due to its unique electrical conductivity, redox properties, low production cost, easy preparation in solution, and good stability at room temperature [13,14]. These properties are crucial in gas sensors as they lower the detection limit, decrease the response time, and improve sensitivity. PANI can be synthesized by the oxidation of the monomer aniline through the chemical oxidative polymerization method [15,16]. In in situ chemical oxidative polymerization, the aniline monomer is oxidized by utilizing ammonium persulfate as the redox initiator, which has been effectively used to deposit the conductive PANI on both conductive and nonconductive substrates [15]. Moreover, it is known that combined PANI with functionalized graphene is an effective way to improve the sensing performance, not only due to the unique properties of graphene but also the combined effect of both materials [17,18,19]. 

It is well accepted that the sensitivities of gas sensors are strongly affected by the specific surface of the sensing materials used, so that a higher specific surface area is directly proportional to the sensitivity and response times of the sensing material [20,21]. Bacterial cellulose (BC), as a special type of cellulose, could be a promising flexible substrate due to its good chemical stability, excellent mechanical strength, and biocompatibility [22,23,24,25]. Research indicates that BC is an excellent supporting material that can be used as deposit nanofillers needed to create advanced BC-based, functional nanomaterials for various technological applications, including gas sensors [25,26,27]. 

This is the first report on the nanopaper composites of cross-linked bacterial cellulose–amino graphene/polyaniline (CLBC-AmG/PANI)-based carbon dioxide (CO_2_) gas sensors. As reported in our previous work, the graphene surfaces were functionalized by using ethylenediamine (NH_2_-(CH_2_)_2_-NH_2_), because it is well known that the amine groups are very sensitive and highly efficient at adsorbing CO_2_ gas [28]. In this work, we designed and fabricated a flexible, freestanding sensor using BC as the flexible substrate and AmG and PANI as active sensing materials. An easy procedure for synthesizing CLBC/AmG nanopaper by the esterification between the carboxyl groups of AmG and hydroxyl groups of BC was reported. In addition, the surface morphology and thermal stability of the CLBC/AmG nanopaper were tested. Then, the PANI was polymerized in situ at the surface of CLBC/AmG nanopaper and the CO_2_ sensing properties of the CLBC-AmG/PANI sensors were investigated and the mechanism of the sensor was discussed.

## 2. Materials and Methods

### 2.1. Materials

Bacterial cellulose (BC) nanopaper was appropriated from Nanonovin Polymer Co. (Mazandaran, Iran). Amino-functionalized graphene (AmG) was synthesized in our lab. Graphene oxide (GO), aniline (ACS reagent, ≥99.5%), *N*,*N*-dimethylformamide (DMF, 99.8%), ethylenediamine (EDA, ≥99%), *N*,*N*′-Dicyclohexylcarbodiimide (DCC, 99%), ammonium persulfate (APS, ≥98.0%), and 5-Sulfosalicylic acid dihydrate (SSA, ≥99%) were all received from Sigma-Aldrich (Oakville, ON, Canada). Deionized (DI) water was used for all the experiments.

### 2.2. Synthesis of CLBC-AmG Nanopaper

**Figure 1:**
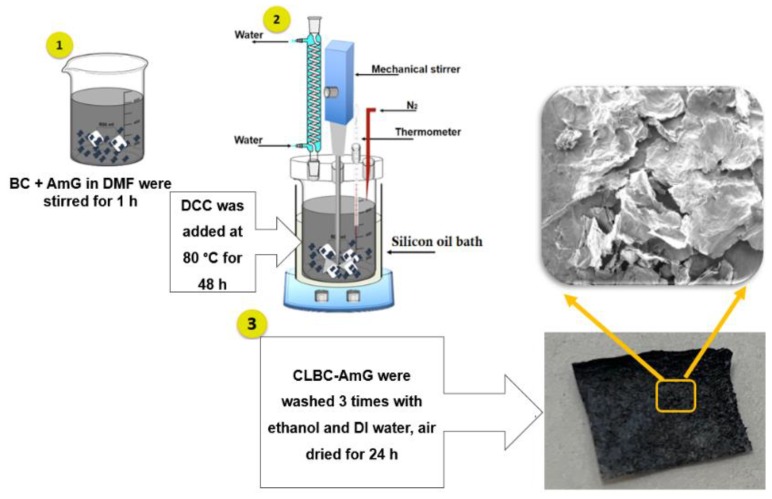
Illustrates the schematic of the fabrication process of cross-linked bacterial cellulose–amino graphene (CLBC-AmG).

As shown in Figure 1, the synthesis of AmG (10 mg) was as reported previously in [28], and BC (50 mg) in DMF (50 ml) were stirred for 1 h. Under vigorous stirring, DCC (100 mg) was added as a dehydration reagent. The esterification between the carboxyl group (-COOH) of AmG and hydroxyl group (-OH) of BC was conducted under N_2_ atmosphere at 80 °C for 48 h to create the crosslinked bacterial cellulose–amino-functionalized graphene (CLBC-AmG). Then the CLBC-AmG fibers were washed several times with ethanol and DI water and then dried under vacuum at room temperature (RT) for 24 h.

### 2.3. Fabrication of CLBC-AmG/PANI Nanopaper Electrodes

**Figure 2:**
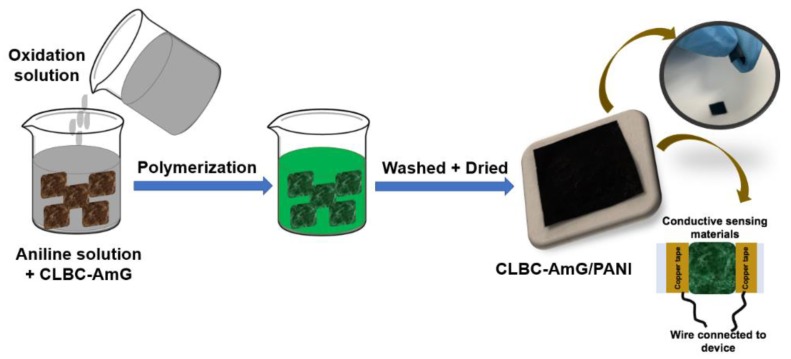
The schematic diagram of the process preparation of This is the first report on the nanopaper composites of cross-linked bacterial cellulose–amino graphene/polyaniline (CLBC-AmG/PANI) flexible electrodes.

The PANI on the surface of CLBC/AmG nanopaper was grown by in situ polymerization. The two solutions were kept for 1 h in the refrigerator at 5 °C before immersing the CLBC/AmG and mixing. The CLBC-AmG nanopaper was immersed in 50 ml DI water of (2.45 g) SSA and (1.86 g) aniline (solution 1). Then 50 ml DI water of (4.45 g) APS (solution 2) was added dropwise into solution 1, which was stirred in an ice-water bath for different polymerization times: 30 min, 1 h, and 2 h. Finally, the period of 30 min was chosen for further studies because it reported more flexibility and good electrical conductivity for sensing purposes. Next, the flexible electrodes of CLBC-AmG/PANI were rinsed three times by DI water and ethanol until the residual oxidant was removed (see Figure 2). For comparison, the BC/PANI electrodes were prepared without AmG by a similar procedure. Finally, the samples were left to dry in air at room temperature (RT). All samples were cut into square pieces (20 × 20 mm) and fixed onto glass slides by applying copper tape to provide the appropriate electrical connection between the sensing substrate and measuring device. At the end, the electrodes were stored at RT under vacuum for two months until the sensing properties were investigated.

### 2.4. Characterization Methods 

A Raman microspectrometer was recorded on a Renishaw InVia Raman microscope (Renishaw, Mississauga, ON, Canada) at an excitation laser wavelength of 514 nm. Thermogravimetric analysis (TGA) was performed using Q5000 TGA (TA instruments, USA) under a nitrogen atmosphere in the temperature range 20–800 °C, with a heating ramp of 10 °C min^−1^. Images using a scanning electron microscope (SEM JSM-7600TFE, FEG-SEM, Calgary, AB, Canada) were collected to study the morphology of the nanocomposites with a very thin layer of (1 nm) gold coating.

### 2.5. Measurement of Gas Sensors

The performance measurements of the fabricated CLBC-AmG/PANI electrodes as a CO_2_ sensor was similar to that which was described in our previous paper. The measurements of the gas sensing properties were tested under laboratory conditions (35–40% relative humidity, RT) using a PalmSens3 (PalmSens EmStat+Potentiostat w/Bluetooth, Compact Electrochemical interfaces, BASi®, West Lafayette, IN, USA) and the mass flow controllers (MFCs) (MKS instruments Inc., 1179C mass-flow®, Kanata, ON, Canada) were used to control the flow rates of the injected gases.

The measurements were obtained using a static process: Initially, the sensor was put into a glass chamber with an inlet and an outlet for gas along with electrical connections. The chamber was first injected with N_2_ via a micro-injector through a rubber plug to measure the initial resistance of the sensor. Then a CO_2_ gas (50–2000 ppm) was injected into the chamber. When the response reaches a constant value, the sensor was exposed to N_2_ to remove CO_2_ and the recovery behavior of the sensor was investigated. 

## 3. Results and Discussion

### 3.1. Characterization of CLBC-AmG and CLBC-AmG/PANI Nanopaper

**Figure 3:**
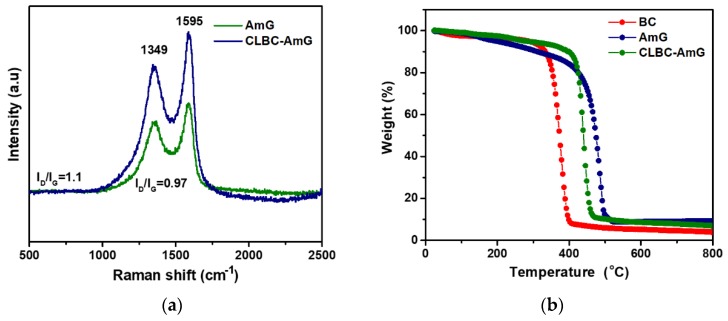
(**a**) Raman spectra of amino-functionalized graphene (AmG) and cross-linked bacterial cellulose–amino graphene (CLBC-AmG) nanopaper. (**b**) Thermogravimetric analysis (TGA) curves of bacterial cellulose (BC), AmG, and CLBC-AmG nanopaper.

The structure of AmG and CLBC-AmG were studied by using Raman spectra, both the AmG and CLBC-AmG nanopaper have two characteristic peaks at 1595 and 1349 cm^−1^ corresponding to the G and D bands, respectively (see Figure 3a) [29,30]. The G band indicates the graphitic structure or whiskers like carbon, whereas the D band refers to the disorder in chemically-functionalized graphene sheets. The intensity ratio of D and G bands (I_D_/I_G_) is used to infer the degree of chemical functionalization in the carbon materials. The CLBC-AmG showed a higher I_D_/I_G_ intensity ratio (1.1) than the AmG (0.97), which is ascribed to BC nanopaper intercalating between the AmG sheets which resulted in increased disorder in the graphene sheets. 

A TGA was conducted to observe the thermal stability of the AmG, BC, and CLBC-AmG. As shown in Figure 3b, the AmG exhibited good thermal stability and large weight loss starts at temperatures of about 449 °C, as the result of the decomposition of amino-carbons, corresponding to previously reported results for the functionalization of graphene with amino groups [28]. The three stages of weight loss can be observed for BC at an initial stage of 35–310 °C, which could be mostly attributed to the moisture evaporation. The major weight loss occurred at the second stage 310–410 °C, and a final weight loss 410–800 °C as the result of degradation and decomposition of the cellulose backbone [29]. In the CLBC-AmG samples, the AmG can considerably improve the thermal stability of BC nanopaper, as shown in Figure 3b.

**Figure 4:**
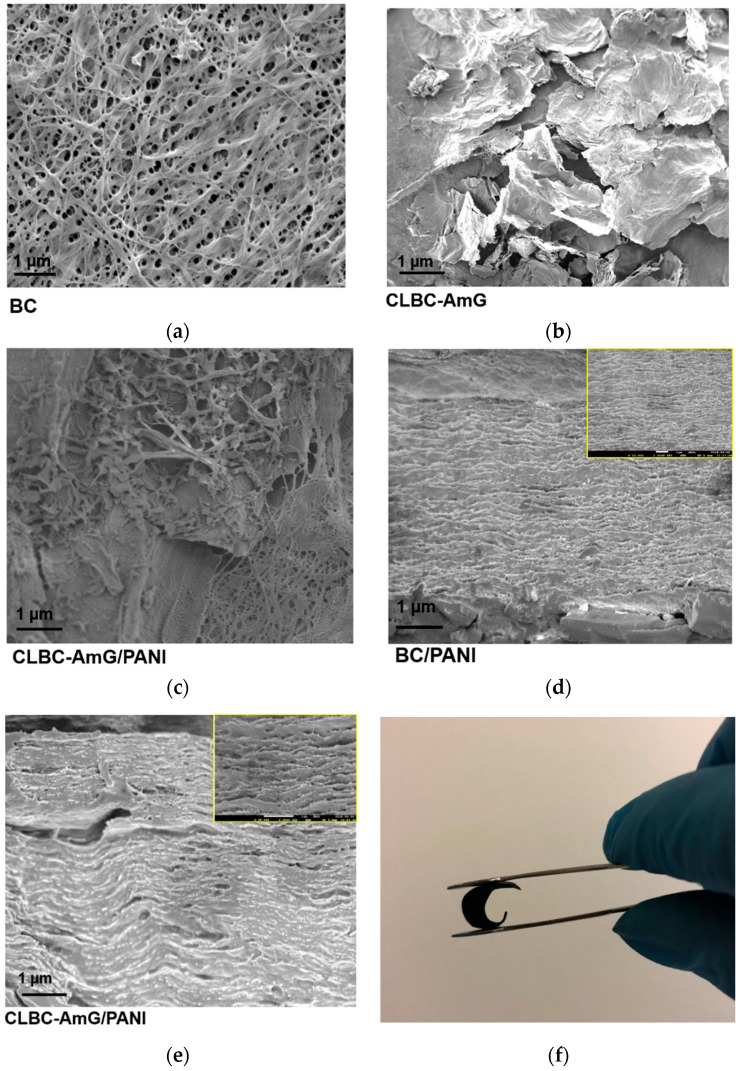
(**a**–**c**) Scanning electron microscope (SEM) micrograph of bacterial cellulose (BC), cross-linked bacterial cellulose–amino graphene (CLBC-AmG), and cross-linked bacterial cellulose–amino graphene/polyaniline (CLBC-AmG/PANI). (**d**,**e**) Cross-sectional SEM images of bacterial cellulose/polyaniline (BC/PANI) and CLBC-AmG/PANI. (**f**) Photograph of CLBC-AmG/PANI nanopaper.

The morphology and structure of BC, CLBC-AmG, and CLBC-AmG/PANI nanopaper are presented in Figure 4a–e. In Figure 4a, the SEM image of BC shows an interconnected, three-dimensional (3D), nanoporous network structure. After the crosslinked BC/AmG via one step esterification, the AmG sheets are clearly interlocked within the 3D web-like arrangement of the BC nanopaper by covalent bonds, as covalent bonding occurs between the reactive groups of both BC and AmG (see Figure 4b). After in situ polymerization, the surfaces of CLBC-AmG are fully covered by hierarchical PANI, which indicates that the PANI are uniformly grown on the surfaces of CLBC-AmG (Figure 4c) [31]. Moreover, the PANI and AmG can be distributed into the BC and forms many channels to provide effective electrolyte transport and active site accessibility, as shown in the cross-section of CLBC-AmG/PANI compared with BC/PANI (see Figure 4d,e) [31]. It should be noted that the freestanding electrode of CLBC-AmG/PANI has good flexibility and can be easily bent, as shown in Figure 4f.

### 3.2. Evaluation and Discussion of Sensor Behavior

We investigated the sensing performance of the CLBC-AmG/PANI for CO_2_ in terms of percentage response, which is defined by the percentile resistance change when the sensors are exposed to CO_2_ as follows: Percentage response = (R_g_ − R_0_)/R_g_ × 100, where R_0_ and R_g_ are the resistances of the sensor before and after exposure to CO_2_, respectively. The response and recovery times of the sensor was defined as the time required to reach 90% of the final resistance.

**Figure 5:**
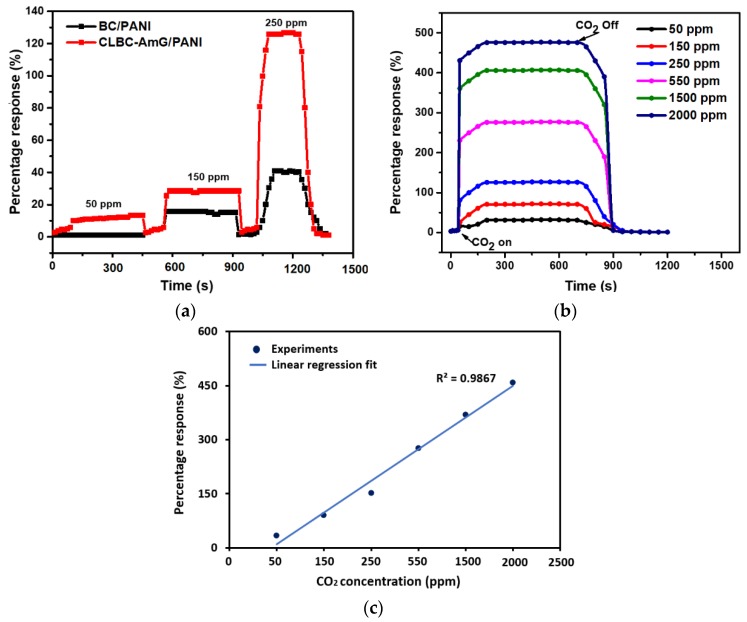
(**a**) Displays the comparison of the dynamic response of the resultant sensors based on bacterial cellulose/polyaniline (BC/PANI) and cross-linked bacterial cellulose–amino graphene/polyaniline (CLBC-AmG/PANI) toward CO_2_ at 50, 150, and 250 ppm concentrations. (**b**) Percentage response of CLBC-AmG/PANI under various concentrations of CO_2_ gas. (**c**) Percentage responses of CLBC-AmG/PANI as a function of CO_2_ concentrations.

**Figure 6:**
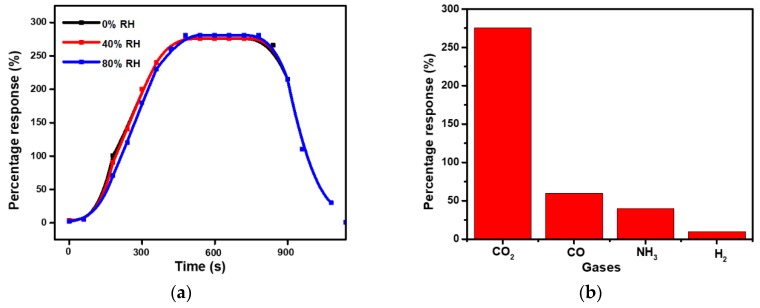
(**a**) Percentage response of cross-linked bacterial cellulose–amino graphene/polyaniline (CLBC-AmG/PANI) exposure to CO_2_ (550 ppm) under different relative humidity (RH) ranges at room temperature (RT). (**b**) Selectivity study of CLBC-AmG/PANI at 550 ppm against other gases.

The performance of the CLBC-AmG/PANI and BC/PANI were studied to verify whether the fabricated CLBC-AmG/PANI have any enhanced CO_2_ sensing properties compared with BC/PANI. Figure 5a shows the response of the sensors to 50, 150, and 250 ppm of CO_2_. It was indicated that the sensors based on CLBC-AmG/PANI nanopaper exhibit resistance increasing, fast and stable response to CO_2_ at RT. It was reported that, upon CO_2_ molecule adsorption, the electrons are released at the p–n junction, which might increase the thickness of the depletion layer [32,33]. Thus, when the thickness of the depletion layer at the interface, between the p-type PANI and n-type AmG in AmG/PANI sensors increased, the resistance also increased [33,34]. Besides, the resistance can be increased due to the reaction between the CO_2_ and primary amine functional groups to form carbamate, where the number of free amines are reduced and subsequently the proton mobility is reduced, which in turn increases the resistance [34]. Furthermore, the CLBC-AmG/PANI sensors were then exposed to various concentrations of CO_2_ gas (50, 150, 250, 550, 1500, and 2000 ppm) and the corresponding response of the sensors were recorded. The sensitivity of the CLBC-AmG/PANI-based CO_2_ sensor was the maximum at 2000 ppm with good response times (~20 s), as shown in Figure 5b. Figure 5c exhibits the sensitivity of the sensor as a function of CO_2_ concentration from 50 to 2000 ppm. The sensor has a wide detection range towards CO_2_ gas: The response greatly increases with the CO_2_ concentration, and is nearly linear with the correlation coefficient close to 0.9867. The limit of quantification (LOQ) of the sensor is defined as the lowest concentration of CO_2_ that can be detected, LOQ = 10 × standard deviation (SD)/slope [35,36]. The detection limit was repeated three times with SD = 2.62. The calculation of LOQ for the CLBC-AmG/PANI sensor is ~26.55 ppm. It was noted that the sensing properties of the CLBC-AmG/PANI sensor toward 550 ppm CO_2_ gas at RT and under humidity levels of 0%, 40%, and 80% relative humidity (RH) were tested (see Figure 6a). No remarkable change in the sensitivity of the CLBC-AmG/PANI sensor with the increase in the relative humidity was observed, yet the response time slightly increased as the relative humidity increased. Moreover, the selectivity is another key factor for the evaluation of a gas sensor, and the results are shown in Figure 6b. The CLBC-AmG/PANI sensors were exposed to various gases of ammonia (NH_3_), hydrogen (H_2_), and carbon monoxide (CO) at 550 ppm. We observed that the response to CO_2_ gas displayed more than thrice the magnitude in comparison with the other analytes. It clearly demonstrates that the sensor CLBC-AmG/PANI nanopaper show an excellent selectivity and can be used as a viable candidate for the detection of CO_2_ gas. 

In our previous publication, we introduced AmG/PANI electrospun nanofiber composites for detecting CO_2_ gas. The device features a chemoresistive sensor that can detect the concentration of CO_2_ accurately. In this sensor, the functionalized graphene with polyaniline as the active material was deposited onto the surface of the electrospun nanofiber substrate of poly(methyl methacrylate) (PMMA). Despite the success, the sensor exhibited less flexibility. In this work, we present a freestanding CO_2_ sensor with excellent flexibility and manageability, a high response and high selectivity to CO_2_ at RT. In addition, it should be noted that the sensing performance of the CLBC-AmG/PANI nanopaper exhibited better sensitivity and fast response time at RT compared with the previously reported CO_2_ sensors, as shown in Table 1. However, the sensor is irreversible and non-reusable at RT. In conclusion, the CO_2_ sensor based on the CLBC-AmG/PANI shows superior flexibility, high selectivity, and accurate detection of CO_2_ concentrations ranging from 50 to 2000 ppm, and this concentration range sufficiently covers the need for CO_2_ detection for many environmental and industrial applications.

## 4. Conclusions

We have developed a sensitive room-temperature CO_2_ gas sensor based on the CLBC-AmG/PANI nanopaper, which was formed by crosslinked BC and AmG via covalent interaction and the PANI was deposited onto the CLBC-AmG surfaces. The CLBC-AmG nanopaper was characterized using SEM, Raman, and TGA techniques. The sensor exhibited a high sensitivity (50 ppm) and selectivity for CO_2_ gas, including superior flexibility and manageability. The sensor responses showed a nearly linear relationship with CO_2_ concentration. Since the preparation process for the CLBC-AmG/PANI sensors was easy and the sensing performance reliable, we believe it has great potential for the sensitive detection of CO_2_ gas in different fields.

## Figures and Tables

**Table 1:** Comparison of sensing performance of our proposed CO_2_ sensor with other published CO_2_ sensors.

Materials.	Range of CO_2_ Concentration (ppm)	Response Time (s)	Temp. (°C)	Ref.
La_2_O_2_CO_3_ nanorods	100–3000	15	325	[37]
La-loaded ZnO	500–5000	90	400	[38]
LaOCL-doped SnO_2_ nanofibers	100–20,000	24	300	[39]
ZnO nanoflakes	200–1025	<20	250	[40]
CLBC-AmG/PANI nanopaper	50–2000	>20	RT	This work
Lanthanum dioxide carbonate (La_2_O_2_CO_3_), lanthanum (La), stannic oxide (SnO_2_), zinc oxide (ZnO), and cross-linked bacterial cellulose–amino graphene/polyaniline (CLBC-AmG/PANI).				
